# Microbiota Composition and Probiotics Supplementations on Sleep Quality—A Systematic Review and Meta-Analysis

**DOI:** 10.3390/clockssleep5040050

**Published:** 2023-12-13

**Authors:** Daniele Santi, Valentina Debbi, Francesco Costantino, Giorgia Spaggiari, Manuela Simoni, Carla Greco, Livio Casarini

**Affiliations:** 1 Unit of Endocrinology, Department of Biomedical, Metabolic and Neural Sciences, University of Modena and Reggio Emilia, 41126 Modena, Italy; daniele.santi@unimore.it (D.S.); valentina.debbi@studenti.unimi.it (V.D.); manuela.simoni@unimore.it (M.S.); livio.casarini@unimore.it (L.C.); 2 Unit of Endocrinology, Department of Medical Specialties, Azienda Ospedaliero-Universitaria of Modena, 41126 Modena, Italy; 3 Unit of Andrology and Sexual Medicine of the Unit of Endocrinology, Department of Medical Specialties, Azienda Ospedaliero-Universitaria of Modena, 41126 Modena, Italy; 4 Center for Genomic Research, University of Modena and Reggio Emilia, 41126 Modena, Italy

**Keywords:** microbiome, probiotics, sleep disorders, sleep quality, gut microbiota

## Abstract

The gut microbiota (GM) plays a crucial role in human health. The bidirectional interaction between GM and the central nervous system may occur via the microbiota–gut–brain axis, possibly regulating the sleep/wake cycle. Recent reports highlight associations between intestinal dysbiosis and sleep disorders, suggesting that probiotics could ameliorate this condition. However, data are poor and inconsistent. The aim of this quantitative metanalytic study is to assess the GM composition in sleep disturbances and evaluate probiotics’ effectiveness for managing sleep disorders. A systematic review was carried out until July 2022 in online databases, limiting the literature research to human studies and English language articles. No significant GM diversity between patients with sleep disturbances versus healthy controls was found, revealed by *α*-diversity, while *β*-diversity is missing due to lack of proper reporting. However, probiotics supplementation significantly reduced the self-assessed parameter of sleep quality and disturbances Pittsburgh Sleep Quality Index (PSQI) score compared with the placebo. No difference in the Epworth Sleepiness Scale (ESS) score was found. While available data suggest that GM diversity is not related to sleep disturbances, probiotics administration strongly improves sleep quality as a subjective perception. However, heterogeneity of data reporting in the scientific literature should be considered as a limitation.

## 1. Introduction

The gut microbiota (GM) is a community of intestinal microorganisms, including *bacteria*, *archaea*, and *eukarya*, constituting the intestinal flora [1]. Physiologically, GM shows (i) metabolic properties, being able to produce essential nutrients, (ii) protective functions through the regulation of mucus production, (iii) structural actions, mediating the expression of tight junction proteins, and (iv) neurological properties, interacting with the peripheral and the central nervous system [2]. It is accepted that GM has broad impacts on human health, impacting the colonization and the resistance to pathogens, maintaining the intestinal epithelium, metabolizing dietary and pharmaceutical compounds, and controlling immune function [3]. The GM composition is heterogeneous and represents an individual signature, reflecting dietary habits [4]. In humans, more than two thousand prokaryotic species distributed in 11 different phyla have been recognized [5,6], among which the large dominant phyla are *Firmicutes* and *Bacteroidetes* [7,8]. Derangement of microbiota may impact its metabolic activities, leading to dysbiosis, which, in turn, could lead to several dysfunctions. Therefore, the evaluation of GM composition gathered increasing relevance [9,10,11,12,13]. GM heterogeneity is described by the α- and β-diversity parameters as measures of species diversity within a community at a local scale and between different communities, respectively [14]. In particular, α-diversity is a measure of microbiome diversity applicable to a single sample, whereas β-diversity quantifies the similarity or dissimilarity between two communities.

In the last decade, the link between GM composition and pathological conditions has gathered increasing interest, suggesting possible connections with infectious and autoimmune diseases, metabolic dysfunctions, neurocognitive disorders, and cancer [15,16,17,18,19,20,21]. The mutual relationship between GM and the host is expected to modulate additional physiological processes, such as sleep, which could be perturbed in the case of dysbiosis [22]. Sleep disturbances are classified into major categories, such as insomnia, sleep-related breathing disorders, central disorders of hypersomnolence, circadian rhythm sleep–wake disorders, sleep-related movement disorders, and parasomnias [23]. Sleep could be described both quantitatively and qualitatively using multiple tools [24]. Polysomnography (PSG) remains the gold standard approach to quality and quantitatively assess sleep, providing detailed information about brain activity, sleep stages, patterns, oxygen saturation, and eye and leg movements [25]. However, PSG is scarcely used in large-scale investigations due to its cost and invasiveness. Alternatively, actigraphy is based on small wrist-watch digital devices monitoring motor activity data for extended periods [26] and is frequently employed in population-based epidemiologic studies [27]. However, it is not recommended for the routine management of sleep disturbances. Thus, several alternative subjective tools, such as direct observations, sleep diaries, and questionnaires, are largely used as well [28]. In particular, validated questionnaires are common tools because of the advantageous cost/benefit ratio despite the less accurate data collection [29]. Among these, the Pittsburgh Sleep Quality Index (PSQI) is a 19-items questionnaire assessing sleep quality [30], while the Epworth Sleepiness Scale (ESS) is an 8-item self-administered questionnaire evaluating the perceived propensity to fall asleep [31,32]. Finally, the nature and severity of insomnia may be assessed by the Insomnia Severity Index (ISI), determined by a 7-item questionnaire [29].

Several pieces of evidence suggest that GM is essential for the maintenance of normal sleep physiology, modulating the production of metabolites involved in sleep homeostasis, such as interleukin (IL)-1β, short-chain fatty acids (SCFAs), serotonin (5-HT), γ-aminobutyric acid (GABA), and melatonin [1,33,34,35]. Experiments in mouse models demonstrated that the SCFA butyric acid modulates the expression of the clock genes *Per2* and *Bmal1*, potentially disrupting circadian rhythms [36,37]. A recent study identified the GM signature of good sleepers, which would be abundant in *Firmicutes*, low in *Prevotella*, and with a high α-diversity index [38]. These data suggest that sleep disorders may be treated by acting on the GM composition using probiotics. They are defined as “live microorganisms which, when administered in adequate amounts, confer a health benefit on the host” [39,40,41] and may be used to treat dysbiosis [39]. Probiotic preparations are largely heterogeneous, and their effects depend, for instance, on dosage, supplementation duration, and bacterial strains [42,43,44]. However, the possible beneficial impact of probiotics on sleep disorders is a matter of debate. In particular, few pieces of evidence are available so far to apply these compounds in clinical practice.

This systematic review and meta-analytic study has two aims: (i) to evaluate differences in GM composition between subjects with sleep disturbances versus healthy controls and (ii) to investigate the effectiveness of probiotics in treating sleep disorders.

## 2. Materials and Methods

The systematic review and meta-analysis were performed according to criteria described in the Preferred Reporting Items for Systematic Reviews and Meta-Analysis (PRISMA) 2015 Statement. Two separate literature searches were carried out from the first available study on probiotics until July 2022, querying the online databases PubMed (MEDLINE), Embase, the Cochrane Library, and Scopus. Then, two different meta-analyses were performed in sequence.

### 2.1. Study Selection and Inclusion Criteria

The first literature search evaluated all published articles concerning the relationship between sleep disorders and GM composition, using the following keywords: microbiota OR microbiome OR (gut microbiota) OR (gut microbiome) OR (gut microflora) OR dysbiosis AND sleep OR (sleep disorder) OR (sleep disturbance) OR (sleep problem). Studies were considered eligible when investigating patients with sleep disorders and reporting GM composition. No restrictions on age, sex, study design (i.e., observational or interventional), year of publication, or tool used to evaluate sleep dysfunctions were applied.

The second literature search was designed to identify all studies in which oral daily supplements containing probiotics (live bacteria) or para-probiotics (heat-inactivated/killed probiotic bacteria) were administered to human subjects with sleep disorders. The following search strategy was applied: probiotic AND sleep OR (sleep disorder) OR (sleep disturbance) OR (sleep problem). Interventional studies in which probiotics or para-probiotics were administered together with other substances were excluded to avoid potential biases. A first screening collected all the interventional studies available, but subsequently, only placebo-controlled studies (probiotic treatment versus placebo) were selected. The randomization was not considered an inclusion criterion.

For both literature searches, two authors (FC and VD) independently collected the eligible articles, and conflicts were resolved by a third investigator (DS). A preliminary screening identified potentially relevant studies based on their titles and/or abstracts. The selected studies were systematically screened for inclusion by full text, according to the aforementioned criteria.

### 2.2. Data Extraction and Analysis

In the first literature search, all extracted data were included in a single dataset, considering authors, year of publication, journal, study design, subjects’ age, patient’s number, microbiota profiling method, sleep assessment methods, criteria for group subdivision according to sleep disorder, *α*–diversity, *β*–diversity, and *Firmicutes* to *Bacteroidetes* ratio, representing a clinical biomarker of dysbiosis [45]. The α-diversity index was considered as the primary endpoint. The main outcome was the comparison of the α-diversity index between subjects with vs. without sleep disorders, as it was the parameter reported across almost all studies, while it was not for *β*–diversity. When studies reported the median and the interquartile range (IQR) of the index, the corresponding mean ± standard deviation (SD) was calculated [46,47].

In the second literature search, the following data types were extracted: authors, year of publication, journal, study design, subjects’ age and body mass index (BMI), patients’ number, microbiota profiling method, sleep assessment methods, intervention(s), probiotic bacteria used and colony forming unit (CFU), placebo treatment, treatment duration, and sleep data (both pre- and post-treatment). Since the most used tool to assess sleep quality was PSQI, it was considered the primary endpoint. The PSQI mean ± SD before and after treatment was used to quantify the probiotics’ effect on sleep quality. When articles reported the standard error of mean (σ), SD was calculated using the following equation:

SEM = σ/√*n*; σ = SEM × √*n* (1)

where n indicates the number of subjects.

Meta-analyses were performed only when more than three studies were available. The parameter indicating heterogeneity among studies (I^2^) was considered as “low,” “moderate,” and “high” for values of 25, 50, and 75%, respectively [48]. Considering the high heterogeneity expected for the outcomes selected, the random effect model was applied to evaluate the mean difference (MD) among continuous data when available. The Review Manager (RevMan) 5.3 software (Version 5.3.1 Copenhagen: The Nordic Cochrane Centre, The Cochrane Collaboration, 2014) was used to perform meta-analyses. When a significant difference was detected by meta-analysis, meta-regression analyses were performed using “Statistical Package for the Social Science” software for Windows (version 28.0; SPSS Inc., Chicago, IL, USA). Statistical significance was considered for *p* values < 0.05.

## 3. Results

### 3.1. Overview of Included Studies

**Figure 1:**
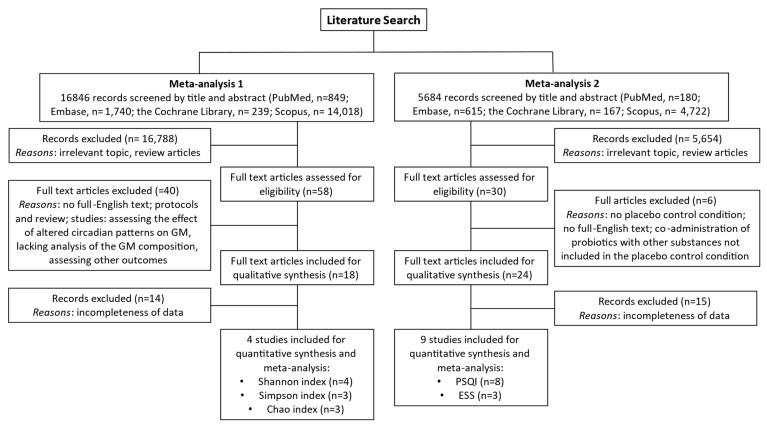
Study flow chart. The relationship between GM composition and sleep disorders (metanalysis 1) and the effect of probiotics on sleep disturbance (metanalysis 2) were assessed.

The first literature search evaluated the relationship between sleep disorders and GM composition. Among 16846 studies detected, fifty-eight potentially relevant studies were extracted, and eighteen articles were finally included (Figure 1). Table 1 summarizes data from studies analyzed during the first literature search.

While not statistically quantifiable, visual assessment of the number of GM components reported in studies shows high variability across studies. The mean age of patients considered was 35.7 ± 22.1 years, ranging from a minimum of 2-year-old children [54] to adults of 68.0 years [62] (Table 1). Similarly, high variability in sleep disorders was detected among studies. Indeed, sleep disturbance pattern includes several conditions of different nature, such as insomnia, reduced sleep quantity, obstructive sleep hypopnea/apnoea syndrome (OSHAS) [54,62], and narcolepsy type 1 [55], not allowing clear conclusions about associations between GM and sleep disturbances (Table 1). Relatively high study heterogeneity is also characterized by the tool used to evaluate sleep quality, which includes PSQI [65], sleep clinical record (SCR) [54], PSG [54,55], ESS [55], and multiple sleep latency test (MSLT) [55], while all studies considered the 16S ribosomal subunit mRNA to profile the microbiota (Table 1).

Eighteen eligible studies [38,49,50,51,52,53,54,55,56,57,58,59,60,61,62,63,64,65] were included in the systematic review, accounting for 236 patients with sleep disorders compared with 233 healthy controls. The α-diversity index was assessed in sixteen of them, while the β-diversity index was assessed in thirteen. However, three different α-diversity indexes were used: the “Shannon” index was evaluated in all the studies, while “Simpson” [55,62,65] and “Chao” [54,55,65] indexes were not. Therefore, α-diversity indexes were used for quantitative analyses. The *Firmicutes/Bacteroidetes* ratio is generally reported, although this is not enough to establish clear differences in sleep patterns in all the populations [54,66,67]. 

The second literature collection included studies describing oral daily supplements of probiotics or para-probiotics administered to individuals with sleep disorders. Among 5684 studies, thirty were evaluated and twenty-four finally included [68,69,70,71,72,73,74,75,76,77,78,79,80,81,82,83,84,85,86,87,88,89,90,91] (Figure 1). Finally, eight studies were eligible to be quantitatively analyzed for PSQI score [68,71,80,87,88,90,91] and three for ESS [87,88,89]. Table 2 summarizes data from studies extracted during the second literature search. 

Overall, 274 subjects treated with probiotics were compared with 261 subjects receiving a placebo. Only one study showed a cross-over design, whereas others employed a randomized approach. Five studies recruited healthy individuals [68,71,78,82], one study [87] enrolled cirrhosis patients recovered from hepatic encephalopathy (HE), one employed [88] patients with irritable bowel syndrome (IBS) and sleep complaints, and one [90] recruited post-menopausal women. Moreover, three studies [71,87,88] reported baseline BMI, and one study enrolled patients with overweight (BMI > 25 kg/m^2^). Regarding the use of probiotics, three studies employed single strains of bacteria [68,78,82] and five multiple strains [71,87,88,90,91] (Table 2). Typically, probiotics were administered within the 108–1011 CFU dose range for a variable duration of 3 [71] to 24 weeks [68,87]. Different media were used to convey the treatment, such as capsules [78,88,91], tablets [68], powder sachets [71,87], fermented milk [82], or yogurt [90], revealing a certain grade of variability among studies for probiotic administration. Compliance with supplementation protocols, if shown, was higher than 95% [82,87,91], except for one study [68], reporting 92%. Thus, as occurred in the first literature search, a high heterogeneity among studies was expected for the aforementioned reasons. Among these, the patient’s compliance with the probiotic consumption protocol should be considered as a further source of heterogeneity. However, none of the studies reported side effects or adverse events, suggesting general good tolerance to the treatment. 

### 3.2. Meta-Analyses

**Figure 2:**
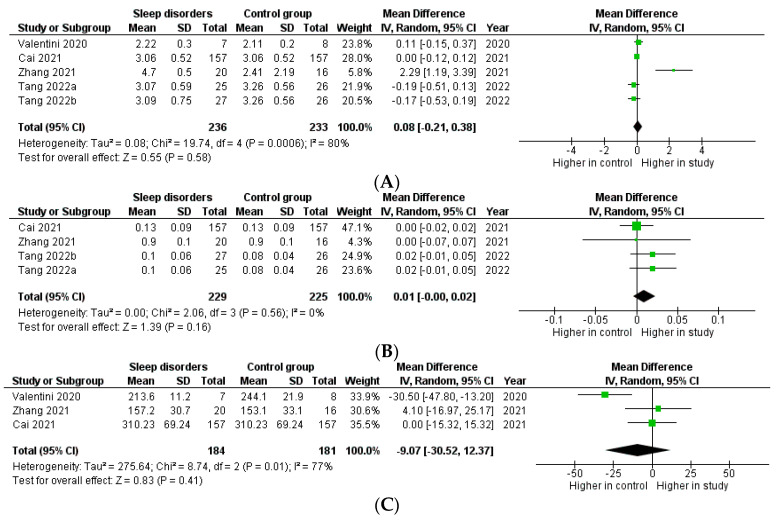
Forest plot for the mean difference of α-diversity. Patients with sleep disorders and healthy controls were compared, considering Shannon (**A**), Simpson (**B**), and Chao indexes (**C**). **References in figure:** Valentini 2020 [54]; Cai 2021 [65]; Zhang 2021 [52]; Tang 2022 [66].

Meta-analysis to compare GM composition between sleep disorders vs. healthy individuals was performed using α-diversity data (Figure 2). α-diversity was not different between patients with and without sleep disorders, considering the Shannon (*p* = 0.580, Figure 2A), Simpson (*p* = 0.160, Figure 2B), and Chao (*p* = 0.410, Figure 2C) indexes. Instead, β-diversity was not considered due to lack of reporting in more than three (i.e., 13) studies.

**Figure 3:**
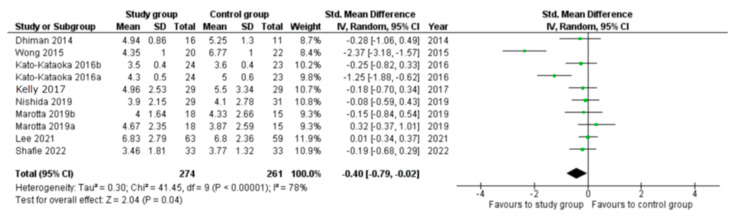
Forrest plot for standard mean difference of PSQI between study (probiotic) and control (placebo) groups. **References in figure:** Dhiman 2014 [87]; Wong 2015 [88]; Kato-Kataoka 2016 [82]; Kelly 2017 [78]; Nishida 2019 [68]; Marotta 2019 [71]; Lee 2021 [91]; Shafie 2022 [90].

For the meta-analytic investigation of probiotics effectiveness in sleep disorders and health, only PSQI and ESS data were accessible in more than three articles. Other sleep measures were not suitable for meta-analysis. Probiotic consumption significantly reduced PSQI score compared with placebo administration (*p* = 0.04, Figure 3). 

Meta-regression analysis was performed using patients’ age as a covariate and the PSQI as the dependent variable. The difference between the study and control group was significantly related to patients’ age (Chi-squared 10.9, *p* = 0.012).

**Figure 4:**
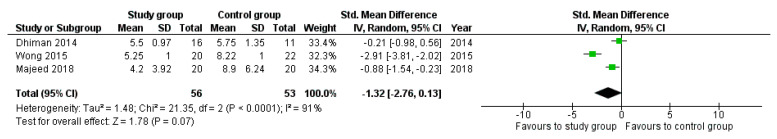
Forrest plot for the standard mean difference of ESS in study vs. control groups. **References in figure:** Dhiman 2014 [87]; Wong 2015 [88]; Majeed 2018 [89].

The use of probiotics was linked to ESS score reduction vs. placebo, although it was not statistically significant (*p* = 0.070, Figure 4). Finally, the mean age of subjects did not differ between the study and control groups (−0.06; 95%CI: −0.05, 0.54 years, *p* = 0.850), excluding the age as a potential source for differences in PSQI score.

## 4. Discussion

This meta-analysis demonstrates that the use of probiotics improves sleep quality, as established by self-assessment questionnaires. However, the self-assessed propensity to fall asleep does not change after probiotics consumption, suggesting that the GM–sleep quality relationship could be only slightly influenced by this therapeutic intervention. Indeed, here we demonstrate that the GM diversity is not different between patients with vs. without sleep disorders, suggesting that there is not a stable substrate on which probiotics could act to improve sleep quality. Results were obtained using α-diversity data, which does not provide a measure of the specific abundance of taxa or species in the gut. Moreover, β-diversity is missing due to a lack of reporting in more than three studies. This is a limitation depending on the heterogeneity of the current literature and precludes the meta-analysis of individual microbiome taxa and/or species. Further, we have considered GM modulation by therapeutic measures on sleep quality as a topic recently explored in a meta-analytics study [92], where authors evaluated the efficacy of probiotic or prebiotic intervention on sleep characteristics. In this study, people with type 2 diabetes mellitus, dementia, and bone fracture were included, and no improvements in sleep quality (eighteen trials analyzed) and sleep duration (five trials analyzed) were found [92]. These results contrast with our results since we found improvement in sleep quality parameters with probiotics administration versus placebo. This discrepancy could be due to the subject analyzed since only healthy subjects or subjects with specific diseases, such as inflammatory bowel disease and fibromyalgia, were included in the present study. Moreover, the authors considered sleep quality parameters using both standardized questionnaires and self-reported information. In the present work, only PSQI reported significant improvement of sleep disturbances compared with the placebo. ESS score seems to be not significantly different, although is shows a trend similar to what observed by PSQI analysis. Unfortunately, heterogeneity of patient’s characteristics with sleep disorders, and wide variability in study experimental settings, make it difficult to generalize further about these results. The role of GM in sleep disorder was recently discussed and converging findings indicated that microbiota could be targeted by interventions aimed at improving sleep [93]. However, we found that sleep disorders could not be associated with GM composition, irrespective to the index used to measure its diversity. On the contrary, PSQI offers the subjective perspective that probiotics administration, obviously influencing GM, improves sleep quality. We may assume that GM could impact sleep behavior, even if possibly weakly, and that this issue requires specific clinical studies with well-selected populations to be addressed.

The scientific interest in the relationship between GM and human functions’ homeostasis has been increasing in recent decades [94,95]. In fact, several lines of evidence indicate the existence of a connection between GM and the central nervous system, leading to the definition of the gut–brain axis (GBA) [96]. The bidirectional flow of information between the GM and the central nervous system is poorly understood and probably works through different mechanisms, such as the hypothalamic pituitary adrenal (HPA) axis [2], the immune system [33,97,98], the intestinal neuroendocrine cells [99], the vagus nerve [100] and the enteric nervous system [2]. The GBA influences sleep status in close interaction with emotions, physiological stress, and circadian rhythms [96]. While the existence of these connections is overall clear, the potential impact of GM on sleep physiology has not been completely unraveled. Sleep patterns could be modulated by changes in intestinal permeability, inflammation, immune system activation, energy harvest, and bacterial diversity [101,102]. However, the relationship between either GM composition or the effect of GM changes after probiotics consumption and sleep patterns remains questionable [103]. Our meta-analysis was not able to quantify the relationship between GM composition and sleep disturbance pattern since no differences in *α*-diversity were found by any indexes used, such as Shannon, Simpson, and Chao. However, taken individually, authors found reduced *α*- and *β*-diversity of GM in specific groups with sleep disturbances [38,51,53,54,57,61]. For example, total GM diversity is positively correlated with increased sleep efficiency and total sleep time when otherwise healthy subjects were considered [104]. Thus, we could speculate that the relationship between GM composition and sleep must be considered, evaluating each group of sleep disturbances separately.

The beneficial effect of probiotics supplementation on sleep disturbance seems to be weak but relatively well established, taking the present study into account with previous analyses [105]. In fact, our findings confirmed that probiotics consumption improves the subjective measure of sleep quality in patients with sleep disturbances when compared with placebo groups. Also, in this case, both sleep disturbance patterns and probiotic composition are extremely variable among studies. Despite this heterogeneity, PSQI is statistically different between the two groups considered, suggesting that the beneficial effect of probiotics could be stronger overall than expected, overcoming potential biases, such as selection and detection biases. Moreover, the mean age of participants is similar between the two groups, excluding the role of age in the observed differential composition of microbial community under probiotics administration. Other subjective sleep questionnaires could be less indicative than PSQI of effects obtained upon probiotics administration. For instance, ESS investigates general parameters such as day time sleepiness, instead of straightly the sleep quality [106]. These considerations might explain why we found no significant improvement in ESS after probiotics administration. However, it is worthy of note that only three studies evaluated ESS [87,88,89], limiting the statistical power of this subgroup meta-analysis. This is a drawback that may be improved by increasing the number of clinical trials on this specific topic. Interestingly, one study was performed on subjects with bipolar disorder, reporting a negative correlation between *Lactobacillus* count and sleep [61]. This finding, obtained in a specific and limited set of subjects, is suggestive of a possible effect of this bacterial strain on sleep health.

It is intriguing that major benefits on sleep were observed among university students exposed to stressful conditions (e.g., academic examinations or courses), as indicated by some of the studies evaluated [68,71,82]. We may speculate that, in these groups, probiotics mitigate markedly the physiological response to stress exposure, via modulation of glucocorticoid action. A perceived environmental threat, such as a stressful event, triggers the HPA axis to secrete glucocorticoids and the sympathetic nervous system, leading to catecholamine release [107]. Moreover, increasing glucocorticoid levels were detected even in short sleep conditions [103]. Some studies indicated that probiotics administration attenuates the physiological increase in salivary glucocorticoids [82,106], potentially promoting sleep enhancement. This hypothesis suggests a beneficial role of probiotics for the general management of sleep disturbances, although subgroup analyses would be useful to establish firm conclusions.

Gender should be considered among potential determinants for high heterogeneity. Women were enrolled in only one study of those considered [90], whereas other trials included mixed groups of both males and females. Since sex-related differences in the effect of probiotics on sleep were suggested [74,84], we may assume that the heterogeneous gender composition of studies meta-analyzed could be a limitation. According to these studies, we could speculate that better outcomes occurred in men than women after *Lactobacillus gasseri* supplementation, although the limited availability of data prevented more accurate evaluations. Second, the participant’s health status varied among trials, including either healthy subjects or individuals affected by different pathological conditions [87,88]. Last, the experimental intervention differed for the type of probiotic bacteria, i.e., species, single vs. multiple strains, dosage, duration of the supplementation, and delivery method. In conclusion, methodological biases should be carefully considered by other studies on this topic.

The present study shows several limitations. The number of trials using the same methodological approach to evaluate sleep disturbance, GM composition, and probiotics type is low. Moreover, studies are highly heterogeneous, including participants with different ages and health statuses and assessment methods to evaluate sleep quality (i.e., subjective or objective measures). In this context, we were not able to collect data useful to adjust the analyses performed. The only available parameter was the patient’s age, which, as demonstrated by meta-regression analysis, potentially influenced the final result of the meta-analysis. Moreover, we could collect only α-diversity data, while β-diversity is missing due to lack of proper reporting, limiting the analysis of how individual microbiome taxa or species may contribute to mitigate sleep disturbances. In particular, the variability in Systematic Reviewpatients’ ages should be carefully considered since it is well-known that sleep patterns undergo physiological changes with aging [108,109]. In our study, the limited number of studies detected does not allow subgroup analyses based on age and gender. Third, the evaluation of sleep quality by questionnaires is obviously influenced by the subjectivity of these instruments. Although self-reported questionnaires are broadly used for clinical and research purposes, they do not provide objective parameters. Thus, data collected could be less accurate than those obtained by objective sleep assessment tools, such as PSG or actigraphy. Finally, only gut microbial genera/species significantly associated with the outcome are likely to be reported in the literature. This would introduce publication bias and limit the number of included studies. Further clinical trials with randomized placebo-controlled designs and objective measurements are required to achieve solid conclusions.

In conclusion, the connection between sleep disturbances and GM homeostasis remains to be established since major drawbacks prevent definitive conclusions about this issue. Promising results based on subjective assessment of sleep quality suggested that probiotics could represent an effective clinical intervention to manage sleep disorders targeting GM. The understanding of variables impacting GM, such as diet, physical activity, stress, and pathological conditions, will be crucial to clarify the relationship between intestinal bacteria and sleep.

## Figures and Tables

**Table 1:** Studies detected during the first literature search.

Common Features						Study Group (Altered)				Control Group (Healthy)		
Authors	Year	Type of Study	Microbiota Profiling Method	Microbiota Detected	Method (Sleep)	Inclusion Criteria	Number	Age (Years ± SD)	Relative Abundance	Number	Age (Years ± SD)	Relative Abundance
Evans	2017 [49]	CCS	16S rRNA amplicon sequencing (V4 region) using Illumina MiSeq	Phyla Bacteroidetes. Firmicutes. Verrucomicrobia. Actinobacteria. Genera Bacteroides. Facealibacterium. Prevotella. Roswburia. Akkermansia. Alistipes. Bifidobacterium. Parabacteroides. Blautia. Phascolarctobacterium. Alistipes	PSQI	BD	115	50.2 ± 12.8	Phylum: Faecalibacterium 5.1 ± 4.3% unclassified Firmicutes 0.6 ± 1%	64	48.6 ± 16.6	Phylum: Faecalibacterium 7.7 ± 5.0%; unclassified Firmicutes 1.1 ± 1.2%
Ko	2019 [50]	CCS	16S rRNA pyrosequencing (V3–V4 regions) using Illumina Miseq	Genera Bacteroides. Ruminococcus. Prevotella	PSG	AHI score > 5 health controls	52	NA	NA	61	NA	NA
Collado	2019 [51]	CCS	16S rRNA amplicon sequencing (V3–V4 region) using MiSeqIllumina protocols	Phylum Tenericutes. Firmicutes. TM7. Lentisphaerae. Fusobacteria. Proteobacteria. Verrucomicrobacteria. Actinobacteria. Bacteroidetes. Porphyromonadaceae. Peptospreptococcaceae and other clostriales	Snoring assessed by interview	Snoring frequency (< or ≥3/week)	27	2.0 ± 0.0	Proteobacteria 1.1%	16	2.0 ± 0.0	Proteobacteria 0.4%
Zhang	2021 [52]	CCS	16S rRNA amplicon sequencing (V4–V5 region) using qIllumina	Phyla Bacteroidetes. Firmicutes Orders Pasteurellales and Actinomycetes Families Bacteroidaceae. Prevotellaceae. Porphyromonadaceae. Rikenellaceae Genera Bacteroides. Prevotella. Parabacteroides. Escherichia. Flavonifractora. Alloprevotella. Parabacteroides. Hungatella	PSQI	MDD diagnosis	36	36.81 ± 13.5	GENUS: Bacteroides 40.0% Prevotella 5.9% Parabacteroides 2.8% Escherichia 2.5% Alistipes 2.2% Alloprevotella 0.5% Tyzzerella 0.3% Paraprevotella 0.2% Haemophilus 0.1% Flavonifractor 0.2% Anaerotruncus 0.1%	45	39.29 ± 11.44	GENUS: Bacteroides 25.0% Prevotella 24.3% Parabacteroides 1.7% Escherichia 0.7% Alistipes 0.8%. Alloprevotella 0.18% Tyzzerella 0.2%. Paraprevotella 0.2% Haemophilus 0.2% Flavonifractor 0.1% Anaerotruncus 0.1% Weissella 0.02. Eisenbergiella 0.01
Fei	2021 [53]	CCS	16S rRNA pyrosequencing (V4 regions) using Illumina Miseq	Family Ruminococcaceae Erysipelotrichaceae Genera Bacteroides. Oscillospira. Catenibacterium. Prevotella. Dialister	Questionnaire	Sleep lenght (short ≤ 7 h. normal 7–9 h long ≥ 9 h)	Short 154 Long 248	Short 35.6 ± 6.2 Long 33.6 ± 6.3	GENUS: Streptococcus 0.7% Coprococcus 1.0% Dorea 0.3 Bamasiella 0.9% Intestinibacter 0.1% SPECIES: Blautia_obeum 0.7% Streptococcus_salivarius 0.7% Clostridium_sp 0.1% Dorea_formicigenerans 0.2% Coprococcus_sp 0.2% Ruminococcus_lactaris 0.7%	250	35.7 ± 6.4	NA
Valentini	2020 [54]	CCS	16S rRNA amplicon sequencing	Phyla Bacteroidetes Actinobacteria Firmicutes Bacteroidetes Proteobacteria Families Clostridiaceae Lactobacillaceae Lachnospiraceae Oscillospiraceae Erysipelotrichaceae Coriobacteriaceae Desulfovibrionaceae Enterobacteriaceae Erwiniaceae Enterobacteriaceae. Erwiniaceae Bacteroidaceae Prevotellaceae Lactobacillaceae Prevotellaceae Sutterellaceae Flavobacteriaceae Genera Colinsella. Eubacterium. Faecalibacterium Colinsella Escherichia Klebsiella Clostridium Lactobacillus Oscillobacter Clostridium Ruminococcus Oscillospira Veillonella Klebsiella	Sleep Clinical Record PSG	OSAHS	7	5.0 ± 1.9	NA	8	8.7 ± 3.6	NA
Zhang	2021 [55]	CCS	16S rRNA amplicon sequencing (V3–V4 region) using Illumina	Order Coriobacteriales Class Coriobacteria Family Barnesiellaceae. Genera Klebsiella. Barnesiella. Ruminiclostridium. Phocea. Blautia. Lactococcus. Bilophila	PSG ESS MSLT	NT1	20	19.0	NA	16	26.0	LDA score: More abundant in HC individuals Class Coriobacteriia 2.26%. Order Coriobacteriales 2.26%. Family Barnesiellaceae 2.52%. Genus Lactococcus 2.37%. Genus Phocea 2.39%. Genus Ruminiclostridium 2.00%. Genus Barnesiella 2.46%. Genus Blautia 3.25%. Genus Bilophila 2.00%
Mercado	2021 [56]	CCS	16s rDNA amplificon sequencing (V3–V4 region) using the MiSeq Illumina	Ezakiella. Clostridium sensu stricto. Porphyromonas and Barnesiella (family Porphyromonadaceae). Coriobacteriales Incertae Sedis. Synergistaceae/Synergistales/Synergistia/Synergistestes. Escherichia-Shigella. Turicibacter	PROMIS-SD	PROMIS-SD. T-score > 55	19 high-occurring symptoms	60.9 ± 16.0	LDA score: More abundant in NT1 individuals Genus Klebsiella 3.19	22 low-occurring symptoms	56.4 ± 7.9	NA
Agrawal	2021 [57]	CCS	16S rRNA amplicon sequencing (V4 region) using Illumina MiSeq	Phyla Firmicutes. Bacteroidetes Order Rhodospirillales Families Acidaminococcaceae. Rikenellaceae. Sutterellaceae.Acidaminococcaceae. Rikenellaceae. Alcaligenaceae.Desulfovibrionaceae. Pseudomonadaceae. Pasteurellaceae Genera Lachnoclostridium. Sutterella. Bilophila. Phascolarctobacterium. Alistipes. Pseudomonas	Sleep length (self-reported)	Sleep length < 6 h short sleepers, 6–8 normal sleepers	16	59.4 ± 7.5	NA	47	62.7 ± 5.8	FIRMICUTES 40% BACTEROIDOTA 36% Lachnoclostridium 1.5% Sutterella 1.25% Alistipes 1.3% Bilophila 0.61% Phascolarctobacterium 0.5% UBA1819 0.13% Paraprevotella 0. 29% Pseudomonas 0.06% Eubacterium_siraeum 0.006%
Hua	2020 [58]	CCS	16S rRNA amplicon sequencing using Illumina MiSeq	Phyla Firmicutes. Actinobacteria. Bacteroidetes. Proteobacteria.Verrucomicrobia Genera Faecalibacterium. Agathobacter	CSHQ	CSHQ < 41	60	4.0 ± 0.2	FIRMICUTES 34% BACTEROIDOTA 39%. Lachnoclostridium 0.40% Sutterella 0.38% Alistipes 0.48% Bilophila 0.25%. Phascolarctobacterium 0.20% UBA1819 0.03% Paraprevotella 0.11% Pseudomonas 0.08% Eubacterium_siraeum 0.13%	60	3.9 ± 0.1	Predominant phyla: *Firmicutes* 43.3%. *Actinobacteria* 28.3%. *Bacteroidetes* 20.7%. *Proteobacteria* 5.6%. *Verrucomicrobia* 1.3%
Buschart	2018 [59]	CCS	16S and 18S rRNA amplicon sequencing (V4 regions) using Illumina HiSeq	Families Corynobacteriaceae. Lachnospiraceae. Rumnococcaceae. Bacteroidaceae. Prevotellaceae. Porphyromonadaceae. Enterobacteriaceae. Phylobacteriaceae. Streptococcaceae. Comamonadaceae. Moraxellaceae.	ESS PSG	PD or iRBD	97 (76 PD and 21 iRBD)	PD: 68.0 ± 9.7 iRBD: 66.1 ± 7.9	Predominant phyla: Firmicutes 43.15% Actinobacteria 25.88% Bacteroidetes 22.57% Proteobacteria 6.34% Verrucomicrobia1.62%	78	68.4 ± 6.7	
Zhang	2020 [60]	CCS	16S rRNA gene sequencing (V4 region)	Phyla Actinobacteria. Proteobacteria. Firmicutes. Bacteroidetes Orders Coriobacteriales. Sphingobacteriales Genera Vagococcus. Adlercreutzia. Bifidobacterium. Parascardovia. Metascardovia. Ruminococcus Species Anaerostipes caccae	OSHAS	OSHAS/OSHAS + cerebral infarction diagnosis	Cerebral infarction group: 28 OSAHS + cerebral infarction group: 28	NA	NA	30	NA	NA
Aizawa	2019 [61]	CCS	16S or 23S rRNA-targeted RT-qPCR	Genera Bifidobacterium. Lactobacillus	HAM-D subscale	BD	39	40.3 ± 9.2	NA	58	43.1 ± 12.9	NP
Tang	2022 [62]	CCS	16S rRNA gene sequencing (V3–V4 region)	Phyla Firmicutes. Proteobacteria Genera Escherichia-Shigella. Faecalibacterium. Streptococcus. Haemophilus. Phascolarctobacterium. Oscillibacter	AHI	OSHAS + T2DM	27	47.6 ± 5.2	NP	26	45.6 ± 8.8	NA
Masyutina	2021 [63]	CCS	16S rRNA gene sequencing	Phyla Actinobacteria Genera Faecalibacterium. Prevotella 9. Lachnospira. Blautia. Faecalibacterium. Lachnospira Species Eubacterium hallii	PSQI ISI	CI diagnosis	55	31.6 ± 7.4	NA	50	33.2 ± 6.6	NA
Grosicki	2020 [38]	CCS	16S rRNA gene sequencing (V3–V4 region)	Phyla Firmicutes. Bacteroidetes. Proteobacteria Classes Clostridia. Clostridia. Negativicutes Orders Clostridiales. Bacteroidales Families Bacteroidales. Lachnospiraceae. Ruminococcaceae Genera Blautia. Prevotella. Faecalibacterium. Ruminococcus. Bacteroides	PSQI	PSQI > 5	9	28.8 ± 10	Euryachaeota 2.41 × 10^4^ Actinobacteria 9.06 × 10^3^ Bacteriodetes 3.16 × 10^1^ Chloroflexi 1.47 × 10^6^ Cyanobacteria 1.84 × 10^3^ Elusimicrobia 1.66 × 10^4^ Firmicutes 5.99 × 10^1^ Fusobacteria 1.36 × 10^4^ Lentisphaerae 6.21 × 10^5^ Proteobacteria 4.19 × 10^2^ Spirochaetes 7.35 × 10^7^ Synergistetes 6.25 × 10^5^ TM7 1.23 × 10^5^ Tenericutes 9.26 × 10^4^ Verrucomicrobia 3.10 × 10^2^ Thermi 2.20 × 10^6^	19	30.3 ± 10.8	Phylum Firmicutes 38.0 ± 10.3 Bacteroidetes 34.6 ± 11.8 Proteobacteria 2.8 ± 1.8 Class Clostridia 32.0 ± 9.9 Bacteroidia 34.6 ± 11.8 Negativicutes 2.2 ± 1.5 Order Clostridiales 32.0 ± 9.9 Bacteroidales 34.6 ± 11.8 Family Bacteroidaceae 13.9 ± 9.3 Lachnospiraceae 9.6 ± 4.2 Ruminococcaceae 12.2 ± 5.5 Genus Blautia 2.2 ± 1.1 Prevotella 16.0 ± 19.1 Faecalibacterium 8.7 ± 4.2 Bacteroides 13.9 ± 9.3 Ruminococcus 2.3 ± 2.6
Bikov	2022 [64]	CCS	16S rRNA gene sequencing (V3–V4 region)	Phyla Actinobacteria. Proteobacteria Class Gammaproteobacteria Families Prevotellaceae. Lactobacillae Genera Porphyromonas. Lachnosporaceae. Lactobacillus. Roseburia	PSG	OSAHS	19	55 ± 12	Phylum Firmicutes 38.0 ± 10.3 Bacteroidetes 34.6 ± 11.8 Proteobacteria 2.8 ± 1.8 Class Clostridia 32.0 ± 9.9 Bacteroidia 34.6 ± 11.8 Negativicutes 2.2 ± 1.5 Order Clostridiales 32.0 ± 9.9 Bacteroidales 34.6 ± 11.8 Family Bacteroidaceae 13.9 ± 9.3 Lachnospiraceae 9.6 ± 4.2 Ruminococcaceae 12.2 ± 5.5 Genus Blautia 2.2 ± 1.1 Prevotella 16.0 ± 19.1 Faecalibacterium 8.7 ± 4.2 Bacteroides 13.9 ± 9.3 Ruminococcus 2.3 ± 2.6	20	43 ± 16	NA
Cai	2021 [65]		rRNA gene sequencing		PSQI	Healthy controls	157	22.3 ± 2.4	NP			

AHI: Apnea-Hypopnea index; ASD: Autism Spectrum Disorder; BD: Bipolar disorder; CSHQ: Children’s Sleep Habits Questionnaire; CCS: case-control study; CI: chronic insomnia; ESS: Epworth Sleepiness Scale; HAM-D: Hamilton Depression Rating scale; ISI: Insomnia Severity Index; MDD: Major Depressive Disorder; MSLT: Multiple Sleep Latency Test; NA: not available; NR: not reported; NT1: Narcolepsy type 1 OSAHS: Obstructive Sleep Apnea/Hypopnea Syndrome; PD: Parkinson disease; PROMIS-SD: PROMIS Sleep Disturbance; PSG: polysomnography; PSQI: Pittsburgh Sleep Quality Index; T2DM: type 2 diabetes mellitus.

**Table 2:** Studies detected during the second literature search.

			Common Features				Study Group (Probiotic)					Control Group (Placebo)		
Authors	Year	Study Design	Microbiota Profiling Method	Sleep Assessment Method	Inclusion Criteria	Treatment Duration	Participants (n)	Age (y ± SD)	Intervention Treatment	Probiotic Bacteria	CFU	Participants (n)	Age (y ± SD)	Placebo Treatment
Nishida et al. [81]	2017	R/DB/PC/P	NP	PSQI EEG	Healthy 6th-year Japanese medical students	12 weeks	34	25.1 ± 2.37	200 mL Fermented Milk/Day	*Heat—inactivated L. gasseri CP2305*	1 × 10^10^	35	25.1 ± 2.4	Milk: high-fructose corn syrup, powdered skim milk, lactic acid, soybean polysaccharide, pectin, sodium citrate, flavors, sweeteners
Kato-Kataoka et al. [82]	2016	R/DB/PC/P	NP	PSQI	Healthy 4th-year medical students undertaking an examination for promotion	6 or 8 weeks	24	23.0 ± 0.4	100 mL Fermented Milk/Day	*L. casei strain Shirota*	1 × 10^9^/mL	23	22.7 ± 0.4	Milk: similar composition with the addition of lactic acid
Nakakita et al. [83]	2016	NR/DB/PC/CO	NP	EEG, AIS, Sleep journals	Healthy males suffering from sleep challenges (AIS ≥ 6)	10 days	6	53.9 ± 8.8	1 × Capsule/Day	*Heat-killed L. brevis SBC8803*	NS	8	53.9 ± 8.8	Capsules: caramel pigment, finely powdered silica, calcium stearate, starch, cellulose
Sawada et al. [84]	2017	R/DB/PC/CO	Gene expression analysis	PSQI	Male medical students undertaking the cadaver dissection course	4 weeks	NS	24	1 × Bag/Day	*L. gasseri CP2305*	1 × 10^10^	NS	24	Lyophilized powder: skim milk (20%), yeast extract (0.50%)
Yamamura et al. [85]	2009	R/DB/PC/CO	NP	Actigraphy, SHRI	Healthy subjects	3 weeks	14	72.1 ± 21.2	100 g Fermented Milk/Day	*L. helveticus*	NS	15	70.7 ± 21.9	Artificially acidified milk added with L-lactic acid
Calandre et al. [86]	2021	R/DB/PC/P	NP	ISI	Fibromyalgia patients	12 weeks	28	56.0 ± 7.5	4 × Sachets/Day	*S. thermophilus BT01* *B. breve BB02* *B. animalis subsp. lactis BL03* *B. animalis subsp. lactis BI04* *L. acidophilus BA05* *L. plantarum BP06* *L. paracasei BP07* *L. helveticus BD08*	4.5 × 10^14^	35	55.5 ± 8.6	Sachets: maltose, cornstarch, silicon dioxide
Dhiman et al. [87]	2014	R/DB/PC/P	NP	PSQI, ESS	Cirrhosis patients	24 weeks	16	48.0 ± 1.4	1 × VSL#3 Sachet/Day	*L. paracasei DSM 24733* *L. plantarum DSM 24730* *L acidophilus DSM 24735*, *L. delbrueckii bulgaricus DSM 24734)* *B. longum DSM 24736* *B. infantis DSM 24737* *B. breve DSM 24732* *Streptococcus thermophilus DSM 24731*	9 × 10^11^	11	50.1 ± 9.8	Sachets: corn flour
Wong et al. [88]	2015	R/DB/PC/P	NP	PSQI, ESS	IBS	6 weeks	20	53.3 ± 18.6	8 Capsules/Day	*L. acidophilus* *L. casei* *L. delbrueckii bulgaricus* *L. plantarum* *B. longum* *B. infantis* *B. breve* *Streptococcus salivarius thermophilus*	1.125 × 10^11^	22	40.9 ± 16.5	Capsules: NS
Majeed et al. [89]	2018	R/DB/PC/P	NP	mESS	MDD and IBS	90 days	20	40.4 ± 10.3	1 × Tablet/Day	*B. coagulans MTCC 5856*	2 × 10^9^	20	43.9 ± 9.8	Tablets: microcrystalline cellulose, starch, sodium starch glycolate, magnesium stearate
Shafie et al. [90]	2022	R/DB/PC/P	NP	PSQI	Post-menopausal women	6 weeks	33	51.8 ± 2.3	100 g Yogurt/Day	*B. lactis* *L. acidophilus*	1 × 10^8^ C CFU/g	33	52.4 ± 2.4	*Yogurt: containing* L. bulgaricus and Streptococcus thermophilus

AIS: Athena Insomnia Scale; CO: cross-over design; DB: double blinded; ECG: electrocardiography; EEG: Electroencephalography; ESS: Epworth sleepiness scale; HE: hepatic encephalopathy; HLPCQ: Healthy Lifestyle and Personal Control Questionnaire; IBS: Irritable Bowel Syndrome; ISI: Insomnia Severity Index; MDD: Major Depressive Disorder; mESS: modified Epworth sleepiness scale; MEQ: Morningness-Eveningness Questionnaire; NP: not performed; NR: non-randomized; OSHAS: obstructive sleep apnea; P: parallel design; PC: placebo-controlled; PCR: polymerase chain reaction; PLA: placebo group; PRO: probiotic group; PSG: Polysomnography; PSQI: Pittsburgh Sleep Quality Index; R: randomized; SD: standard deviation; SHRI: Sleep-Health Risk Index; VAS: Visual analog scales. Italics are used for bacterial and viral taxa at the level of family and below.

## Data Availability

Data reviewed and analyzed during this meta-analysis is available from the corresponding author upon reasonable request.

## List of Abbreviations

BMI, body mass index; CFU, colony forming unit; ESS, Epworth Sleepiness Scale; GABA, γ-aminobutyric acid; GBA, gut–brain axis; GM, gut microbiota; HE, hepatic encephalopathy; HPA, hypothalamic pituitary adrenal; 5-HT, serotonin; IBS, irritable bowel syndrome; IL, interleukin; IQR, interquartile range; MD, mean difference; MSLT, multiple sleep latency test; OSHAS, obstructive sleep hypopnea/apnoea syndrome; PSG, polysomnography; PRISMA, Preferred Reporting Items for Systematic Reviews and Meta-Analysis; PSQI, Pittsburgh Sleep Quality Index; SCFAs, short chain fatty acids; SCR, sleep clinical record; SD, standard deviation.
